# High-Performance Room-Temperature Terahertz Photodetection Using 2-Dimensional Electron Gas Channel Transport

**DOI:** 10.34133/research.0656

**Published:** 2025-03-26

**Authors:** Mengjuan Liu, Yongzhen Li, Ziyang Ren, Yao Wang, Haiming Zhu, Qinxi Qiu, Nasir Ali, He Zhu, Jiaqi Zhu, Weien Lai, Zhiming Huang, Huizhen Wu

**Affiliations:** ^1^ Zhejiang Key Laboratory of Micro-nano Quantum Chips and Quantum Control, School of Physics, and State Key Laboratory of Silicon and Advanced Semiconductor Materials, Zhejiang University, Hangzhou 310058, China.; ^2^ State Key Laboratory of Infrared Physics, Shanghai Institute of Technical Physics, Chinese Academy of Sciences, Shanghai 200083, China.; ^3^ University of Chinese Academy of Sciences, Chinese Academy of Sciences, Beijing 100049, China.; ^4^ Computing Research Center for Intelligent Manufacturing, Zhejiang Lab, Yuhang District, Hangzhou, Zhejiang 311121, China.; ^5^ Hangzhou Institute for Advanced Study, University of Chinese Academy of Sciences, Hangzhou, Zhejiang 310024, China.; ^6^ School of Instrument Science and Opto-electronics Engineering, Hefei University of Technology, Hefei, Anhui 230009, China.

## Abstract

Room-temperature (RT) terahertz (THz) detection finds widespread applications in security inspection, communication, biomedical imaging, and scientific research. However, the state-of-the-art detection strategies are still limited by issues such as low sensitivity, narrow response range, slow response speed, complex fabrication techniques, and difficulties in scaling up to large arrays. Here, we present a high-sensitivity, broadband-response, and high-speed RT THz detection strategy by utilizing a deep subwavelength metal–semiconductor–metal (MSM) structure. The spontaneously formed 2-dimensional electron gas (2DEG) at the CdTe/PbTe interface provides a superior transport channel characterized by high carrier concentration, low scattering, and high mobility. The synergy of the electromagnetic induced well effect formed in the MSM structure, and the efficient and rapid transport capabilities of the 2DEG channel give rise to an impressive performance improvement. The proposed 2DEG photodetector exhibits a broad frequency range from 22 to 519 GHz, an ultralow noise equivalent power of 3.0 × 10^−14^ W Hz^−1/2^ at 166 GHz, and a short response time of 6.7 μs. This work provides an effective route for the development of high-performance RT THz detection strategies, paving the way for enhanced THz technology applications.

## Introduction

Owing to its broadband nature, unique spectral fingerprint, safety, and penetration capabilities, terahertz (THz) waveband (0.1 to 10 THz) holds promise for diverse applications, including biomedical effects [1,2], nondestructive testing [3,4], communications [5], and aerospace technologies [6,7]. Despite extensive research being conducted on various strategies for room-temperature (RT) THz detection, they struggle with trade-offs between different figures of merit such as sensitivity, response speed, operating frequency range, and scalability for large-scale arrays. Thermal detection strategies like pyroelectrics, bolometers, and Golay tubes cover a broad operating frequency range but suffer from inferior noise equivalent power (NEP) (10^−9^ to 10^−10^ W Hz^−1/2^) and long response time (10^−2^ to 10^−3^ s) [8–10]. Traditional photoelectric detection strategies of photoexcitation transitions are limited by low photon energy of THz wave and interference from the background radiations in the surrounding environment. Schottky barrier diodes (SBDs), achieving electrical rectification THz detection based on nonlinear current–voltage (*I*–*V*) characteristics, offer improved sensitivity (NEP ~10^−12^ W Hz^−1/2^) but narrow operating range [11–14]. Not only that, to match the rapid oscillation speed of the THz wave, the junction distance needs to be minimized, which can induce undesired junction parasitic capacitance and consequently restricts further sensitivity improvement.

Innovative strategies are rapidly advancing in RT THz detection focusing on field effect transistors (FETs) and high electron mobility transistors (HEMTs) [15–20]. Notable examples include Si FET (NEP ~10^−11^ W Hz^−1/2^) and GaN/AlGaN HEMT (NEP ~10^−12^ W Hz^−1/2^). These devices typically require multi-electrode configurations with nanoscale channel sizes, unfavorable for fabrication and large-scale array. Recently, the emerging surface plasmon polariton (SPP)-induced detection strategy demonstrated high-sensitivity and rapid response capabilities [21,22]. However, the fabrication processes of nanogroove microstructure and spiral antenna demanded by the device are intricate, so this leads to limit their potential for large-scale array application. In addition, some promising materials, such as black phosphorus (BP), graphene, and Weyl semimetals, are currently under investigation for RT THz detection based on photothermoelectric (PTE), intraband transition, or topological effect mechanisms (NEP ~10^−11^ W Hz^−1/2^), yet the immature material growth technologies limit their further improvements and applications [23–33].

A recently developed RT THz detection strategy, known as the electromagnetic induced well (EIW) effect, has drawn attention for its use of a simple deep subwavelength metal–semiconductor–metal (MSM) structure, enabling an optoelectronic response beyond the semiconductor bandgap [34,35]. When an electromagnetic wave with photon energy much smaller than the bandgap of the semiconductor strikes the MSM structure, an EIW forms in the semiconductor. This EIW captures carriers originating from the metal, modifies the conductivity of the semiconductor, and allows the photocurrent signal of the electromagnetic wave to be detected [36–38].

Herein, we propose an RT THz photodetector with high figures of merit including sensitivity, response speed, and operational frequency range based on an epitaxially grown CdTe/PbTe (111) semiconductor heterojunction using an MSM physical structure. Due to the EIW effect, nonequilibrium electrons are emitted into the semiconductor when THz wave irradiates on the device. The mismatch in the bonding coordination at the heterojunction interface induces a 2-dimensional electron gas (2DEG) here, which acts as the transport channel of the nonequilibrium electrons. Benefitting from the high density and the high mobility of the 2DEG, the nonequilibrium electrons would be collected with high efficiency and high speed under biased voltage. Therefore, both the sensitivity and the response speed are distinctly improved by applying the 2DEG transport channel. The 2DEG photodetector achieves the best NEP of 3.0 × 10^−14^ W Hz^−1/2^ at 166 GHz and a response time of 6.7 μs. Additionally, the response frequency range is substantially broadened to 22 to 519 GHz. This amazing improvement caused by the 2DEG transport channel contributes to the development of high-performance RT THz detection strategies.

## Results and Discussion

### Properties of PbTe and CdTe/PbTe materials

**Fig. 1.**
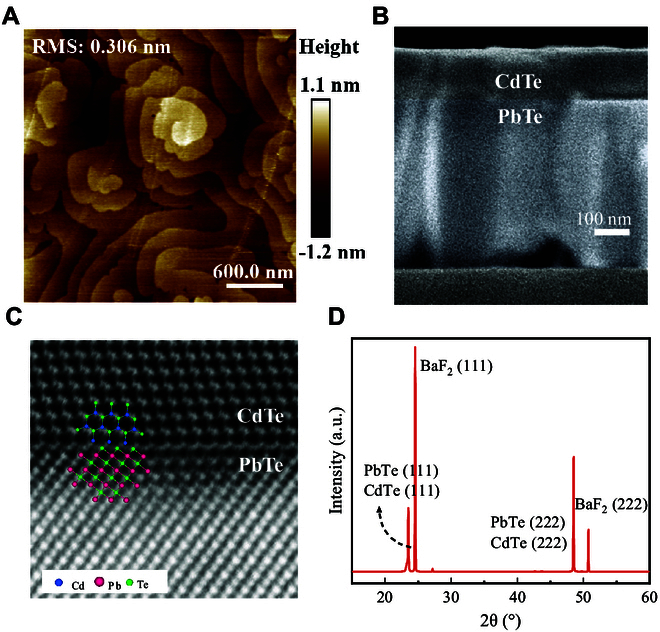
Structural and morphological analysis of the grown materials. (A) AFM image of the PbTe surface grown by MBE on the BaF_2_ substrate. (B) Cross-sectional SEM image of the CdTe/PbTe heterojunction. (C) HRTEM image of the CdTe/PbTe heterojunction interface. (D) XRD pattern of the CdTe/PbTe heterojunction.

Using molecular beam epitaxy (MBE) technology, two 500-nm-thick PbTe bulk films were grown on 2 freshly cleaved BaF_2_ (111) substrates, and a 100-nm-thick CdTe layer was then deposited on top of one of the as-grown PbTe films to form a CdTe/PbTe (111) heterojunction. The RT electrical properties of the epitaxially grown PbTe film and CdTe/PbTe heterojunction materials were characterized using the Hall effect based on the Van der Pauw method (see Fig. S1). The PbTe film exhibits a p-type electrical conductivity with a hole density of 1.2 × 10^13^ cm^−2^ and carrier mobility of 459 cm^2^/V·s, while the CdTe/PbTe heterojunction exhibits the inverse conductivity type with a much higher electron density of 9.8 × 10^14^ cm^−2^ and a higher carrier mobility of 760 cm^2^/V·s. The important transition from film to heterojunction is governed by the formation of a 2DEG at the interface, which largely determines the electrical properties in the heterojunction [39]. Several characterization technologies were employed to analyze the surface morphology, cross-section, and crystallinity of the grown materials. Figure 1A presents an atomic force microscopy (AFM) image of the PbTe layer surface. The root mean square (RMS) roughness of the film surface is 0.306 nm, which is comparable to the thickness of a single atomic layer (0.373 nm). Moreover, the AFM image further reveals a clear helical structure on the sample surface. This morphology is attributed to dislocation lines at the PbTe/BaF_2_ interface spiraling upward with increasing film thickness. This phenomenon is indicative of a unique dislocation-regulated epitaxial growth mode of PbTe on BaF_2_, which promotes line dislocation clustering and thus reduces the overall dislocation density. Figure 1B shows a cross-sectional scanning electron microscopy (SEM) image of the CdTe/PbTe heterojunction, confirming the designed thicknesses of the PbTe (500 nm) and CdTe (100 nm) layers. Similarly, Fig. 1C shows a high-resolution transmission electron microscopy (HRTEM) image of the CdTe/PbTe heterojunction interface, clearly revealing the atomic arrangement near the interface. The image suggests that the epitaxially grown heterojunction has a clean and well-defined interface. It can be observed that CdTe and PbTe share a common interface monolayer of Te atoms. The interface Te atoms receive an excess of valence electrons from both the Cd atoms in CdTe and the Pb atoms in PbTe. In the simplest consideration based on electron counting, i.e., assuming full ionicity and no charge redistribution, on average, the Te atom should receive 1.0 electron (6p) from the 3 Pb atoms and 1.5 electron (5s) from the 3 Cd atoms, implying an excess of valence electrons with total 8.5 valence electrons per interface Te atom, thus spontaneously yielding a metallic interfacial layer (2DEG) [40]. The 2DEG channel forms spontaneously at the interface during the epitaxial growth process rather than through intentional doping, resulting in low scattering and very high electron mobility in the channel. Additionally, x-ray diffraction (XRD) measurement was also performed on the CdTe/PbTe heterojunction using Cu K_α_ radiation in the reflection configuration, and the XRD pattern is shown in Fig. 1D. The intense diffraction peaks indicate that both PbTe and CdTe layers are oriented along the [111] direction. The lattice constants of the PbTe and CdTe films were calculated to be 6.450 and 6.504 Å, respectively, using the Bragg diffraction formula and the crystal plane spacing formula (see Fig. S2). This reveals the small lattice mismatch at the interface, which results in the achievement of the perfect interface of the CdTe/PbTe heterojunction as observed in Fig. 1C.

### Operation principle for RT THz detection

**Fig. 2.**
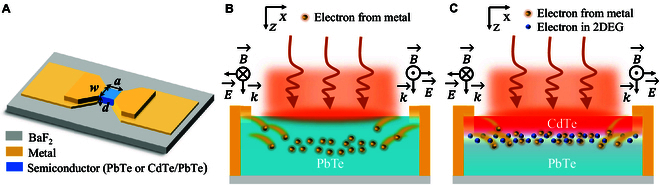
Working mechanisms of the THz photodetectors with and without 2DEG. (A) Schematic diagram of a THz photodetector with a subwavelength MSM structure, showing the length (*a*), width (*w*), and thickness (*d*) of the photosensitive semiconductor region, with metal components acting as electrodes. (B) The working mechanism of the PbTe THz photodetector, where the THz wave interacts with the MSM structure, causes the Lorentz force to drive electrons from the metal electrodes into the PbTe film. (C) Working mechanism of the CdTe/PbTe 2DEG THz photodetector, where the electrons from metal electrodes enter into the 2DEG channel at the interface.

As shown in Fig. 2A, the fabricated devices based on the grown PbTe and CdTe/PbTe for RT THz detection were composed of a simple deep subwavelength MSM physical structure. Figure 2B displays the dynamic evolution caused by EIW effect occurring in the PbTe photodetector under THz wave irradiation. When the MSM structure is irradiated with THz waves, the incident antisymmetric electric field induces a potential well in the semiconductor during the first half of the cycle. As a result, the Lorentz force drives electrons from the metal electrodes into the PbTe semiconductor, where they become confined within the EIW [34,35]. In the following second half of the cycle, the incident electric field creates a potential barrier that slows down the emitted electrons from the metal electrodes. Consequently, these nonequilibrium electrons are trapped in the potential, altering its electrical conductivity under THz wave irradiation. A detailed theoretical derivation of this process is given in Fig. S3. This phenomenon is similar to the well-known photoconduction effect in visible and infrared wavebands, and hence, we call it “abnormal photoconduction”, which is also the so-called EIW effect. When the electrodes are voltage-biased, photocurrent is naturally generated, suggesting that the incident optical signal is successfully converted into an electrical signal. Since abnormal photoconduction is not related to energy level transitions, the detectable photon energy of this kind of photodetector can reach far below the semiconductor bandgap with a little background radiation interference.

Next, we fabricate a device with identical dimensions using CdTe/PbTe, an extraordinary heterostructure with 2DEG. Metal Cu atoms infiltrate into 2DEG channel forming ohmic contact. An electromagnetic-induced well will be formed in the detector when THz waves irradiate at the MSM structure. Similarly, the nonequilibrium electrons are emitted into the 2DEG channel by the Lorentz force, as displayed in Fig. 2C. Due to the band structure of the 2DEG at the heterojunction interface crossing the Fermi level, electrons can easily enter the conduction band of the 2DEG directly [41] (see Fig. S4). In this case, the 2DEG acts as the transport channel for nonequilibrium electrons, replacing the semiconductor. The high electron concentration of the 2DEG markedly reduces the recombination rate of these nonequilibrium electrons, thereby greatly enhancing their collection efficiency. Besides, benefiting from the spontaneously formed 2DEG at the CdTe/PbTe interface without intentional doping, electrons in the 2DEG channel exhibit low scattering and higher carrier mobility. The higher carrier mobility of the 2DEG compared with the semiconductor enhances the transport speed in the channel, thus increasing the response speed of the photodetector. Moreover, the electron lifetime in the 2DEG channel is much higher than that in the semiconductor, resulting in a higher photoconductive gain in the photodetector. Therefore, the overall performance is reasonably improved by introducing the 2DEG transport into the RT THz detection.

### Photoelectric performance comparison

**Fig. 3.**
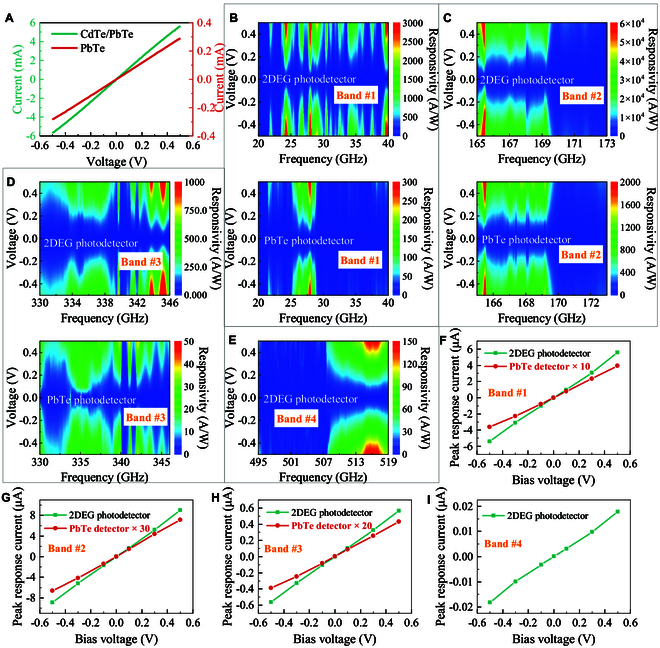
Photoelectric response characterization. (A) *I*–*V* characteristic curves of the 2 photodetectors. (B to E) Response spectra of 2 photodetectors in a microwave source (20 to 40 GHz: band #1) and 3-THz wave sources (165 to 173 GHz: band #2, 330 to 346 GHz: band #3, and 495 to 519 GHz: band #4) under various bias voltages. The PbTe photodetector can respond to all bands except band #4, while the 2DEG photodetector can respond to all above bands. (F to I) Highest response current in respective bands of the 2 photodetectors, where the photocurrents of 2DEG photodetector are enhanced by 14.2, 37.7, and 25.9 times that of the PbTe photodetector at bands #1, #2, and #3, respectively.

We characterized the photoelectric performance of the PbTe and the 2DEG THz photodetectors at RT and ambient environments. As shown in Fig. 3A, under dark conditions, both *I*–*V* characteristics of the 2 photodetectors show fine linearity, indicating a good ohmic contact between the PbTe or the 2DEG and the electrodes. In the PbTe photodetector, the PbTe and the electrodes are in close physical contact, and thus, the ohmic contact can be easily realized as long as their work functions are well matching, while the situation is different in the 2DEG photodetector. As schematized in Fig. 2C, the 2DEG and the electrodes are spatially insulated by the CdTe. Consequently, thermal annealing is required for the diffusion of the metal through the CdTe, contacting the 2DEG channel. To characterize the response spectra of the 2 photodetectors, a microwave source (20 to 40 GHz: band #1) and 3-THz wave sources (165 to 173 GHz: band #2, 330 to 346 GHz: band #3, and 495 to 519 GHz: band #4) were utilized successively, and the measured photocurrent spectra were all normalized by the emitted power spectra of the sources to get the responsivity spectra. As displayed in Fig. 3B to E, the PbTe photodetector can respond to all bands except band #4, while the 2DEG photodetector can respond to all bands, indicating that applying the 2DEG channel in RT THz detection can notably broaden the response frequency range. In essence, the 2DEG channel improves the collection efficiency and the photoconductive gain, and hence, the signal-to-noise ratio (SNR) in band #4 is markedly increased from the undetectable level of the PbTe photodetector to the obtained level of the 2DEG photodetector. This improvement is more directly embodied in bands #1, #2, and #3, where the spectral responsivities are all enhanced by respective ratios. With varying biased voltage, the responsivity distributions in every band remain almost unchanged for the 2 photodetectors, following the photoconductive property. The highest response currents as a function of biased voltage in the respective bands of the 2 photodetectors are presented in Fig. 3F to I. In these bands, the photocurrents of the 2DEG photodetector are enhanced by factors of 14.2, 37.7, and 25.9 compared to those of the PbTe photodetector at bands #1, #2, and #3, respectively. All the curves show a clear linear relation, solidly verifying that our photodetectors are photoconductive type.

**Fig. 4.**
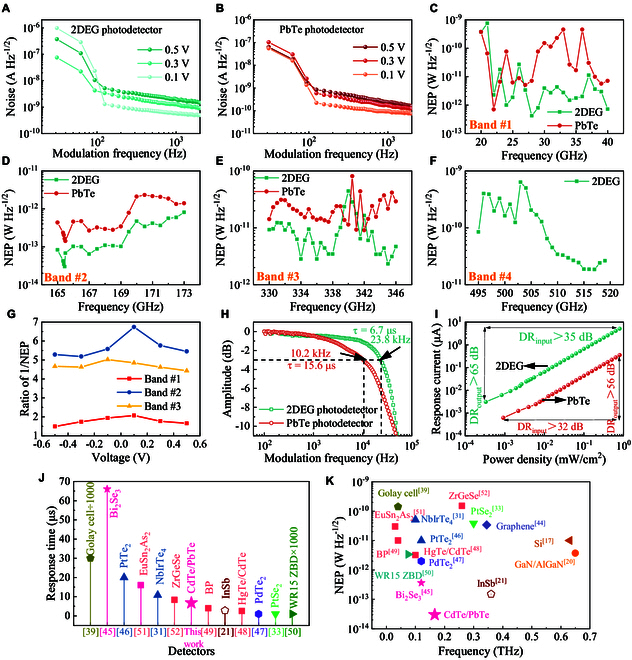
Evaluation for figures of merit. (A and B) Current-noise spectra of the 2 photodetectors under different biased voltages. (C to F) NEP spectra of the devices across different frequency bands at 0.5 V. The NEP levels of the 2DEG photodetector are obviously lower than those of the PbTe photodetector. (G) Ratios of 1/NEP at bands #1, #2, and #3. (H) Amplitude–frequency responses of photodetectors with or without 2DEG, corresponding to the response time of 6.7 and 15.6 μs, respectively. (I) Input and output LDRs of 2 photodetectors. (J and K) Comparisons of response time and NEP of 2DEG photodetectors with other RT THz detectors [17,20,21,31,33,39,44–52].

To explore the electric noise property of the 2 photodetectors, we also measured their current-noise spectra in the frequency domain under different biased voltages, as given in Fig. 4A and B. In these noise spectra, 1/*f* noise dominates the total noise at low modulated frequency, where it declines sharply with an increase in modulated frequency, while as modulated frequency reaches above 125 Hz, shot noise becomes dominated. The modulated frequency during the spectral response characterizations is selected as 500 Hz, ensuring that shot noise is dominated when the 2 photodetectors operate. In photoconductors, shot noise originates from carrier fluctuations within the transport channel, such as hole fluctuations in PbTe or electron fluctuations in the 2DEG of our photodetectors. The level of this noise depends on both the biased voltage and the carrier concentration. Higher biased voltage promotes the fluctuation intensity, hence raising the shot noise, as shown by the noise spectra (Fig. 4A and B). Also, higher carrier concentration leads to more carriers participating in the fluctuation behavior such that the 2DEG photodetector presents a higher noise level compared with the PbTe one. It is undeniable that this approach inevitably suffers from increased noise due to the 2DEG participating in transport.

In terms of the responsivities and the noise spectra, NEP of the 2 photodetectors can be attained using the formula NEP = *i_n_/R_i_*, where *i_n_* is the current noise and *R_i_* is the responsivity. The NEP spectra at 0.5 V in different bands are plotted in Fig. 4C to F. The NEP levels of the 2DEG photodetector are all suppressed clearly compared with the PbTe photodetector in bands #1, #2, and #3. The 2DEG photodetector achieves the optimal NEPs in the 4 bands, which are 4.2 × 10^−13^ W/Hz^1/2^ (at 28 GHz), 3.0 × 10^−14^ W/Hz^1/2^ (at 166 GHz), 2.0 × 10^−12^ W/Hz^1/2^ (at 345 GHz), and 1.8 × 10^−11^ W/Hz^1/2^ (at 516 GHz), respectively. The optimal NEPs versus biased voltage in respective bands of 2 photodetectors are given in Fig. S5. Figure 4G shows the optimized ratios of the best NEPs obtained by applying the 2DEG channel in different bands. Because of the high improvement of responsivity, the ratios of ~5 times are achieved in bands #2 and #3, although the noise increase restrains the optimization. Imperfectly, the optimization ratio drops to ~2 times in band #1. The noteworthy optimization effect on NEP verifies the advantages of the 2DEG channel in transporting nonequilibrium carriers.

Response speed is another important figure of merit for evaluating the photodetector performance. Figure 4H presents the curves of the normalized response intensities as a function of the modulated frequency of the 2 photodetectors. The −3-dB bandwidth (*f*_−3dB_) of PbTe and 2DEG photodetectors is 10.2 and 23.8 kHz, respectively, and the response time obtained using the formula *t*_r_ = 1/2π*f*_−3dB_ is 15.6 and 6.7 μs, respectively. This means that the response speed is increased by more than 2 times with the utilization of the 2DEG channel to transport photogenerated carriers, which benefitted from higher mobility in the 2DEG. Moreover, we also studied the linear dynamic range (LDR) performance of the 2 photodetectors, and the measured results are displayed in Fig. 4I. Since the 2DEG photodetector possesses a lower NEP than the PbTe photodetector, the lower limit of the LDR is also smaller. Unfortunately, our experimental setup could only produce a maximum power density of 0.8 mW/cm^2^, which remains within the LDR. Consequently, we were unable to precisely determine the upper limit of the LDR. Nonetheless, the input and output LDR values were measured as 32 and 56 dB for the PbTe photodetector, respectively, and 35 and 65 dB for the 2DEG photodetector, respectively. The 16% improvement in LDR markedly broadens the range of application scenarios, making it suitable for nearly all practical conditions. The performance improvement on NEP, response time, and LDR deriving from the 2DEG channel transport exhibits a promising future for our strategy in RT THz detection.

Afterward, the response time and NEP of the 2DEG photodetector are compared with other kinds of state-of-the-art RT THz detectors, as displayed in Fig. 4J and K. More details (operation mechanism, temperature, frequency band, responsivity, detectivity, and fabrication complexity) are given in Table S1. Figure 4J and K intuitively depicts the fast response and high sensitivity of the 2DEG photodetector due to the proposed 2DEG transport channel. Compared to other RT THz detectors, the 2DEG photodetector is at the forefront in terms of rapid response performance, and the sensitivity is nearly the best. The proposed RT THz detection implementation exhibits substantial advantages due to its superior performance and simple physical structure. This work paves the way for utilizing such semiconductor materials with a 2DEG to develop photodetectors that surpass current standards in comprehensive figures of merit.

## Conclusion

This study successfully demonstrates a high-performance RT THz photodetector combining the EIW effect with the 2DEG transport channel, utilizing a simple MSM physical structure. The undoped 2DEG at the CdTe/PbTe interface provides a superior transport channel with high carrier concentration, reduced scattering, and enhanced mobility. By employing the 2DEG channel, the recombination of nonequilibrium electrons is notably reduced before being collected, and the transport speed is also faster, thereby improving responsivity and response speed of the 2DEG photodetector compared with the PbTe one. The electron lifetime in the 2DEG channel is longer than that in the semiconductor, leading to an increased photoconductive gain in the photodetector. Specifically, the highest responsivity of the 2DEG photodetector is up to 37.7 times higher, and the response time is 2.3 times shorter than that of the PbTe photodetector. The utilization of the 2DEG transport channel remarkably enhances the response of the photodetector. Despite the increased shot noise resulting from the higher electron concentration, the 2DEG photodetector maintains a higher sensitivity. Therefore, the 2DEG photodetector exhibits an exceptionally low NEP of 3.0 × 10^−14^ W Hz^−1/2^ at 166 GHz, with a sensitivity improvement of 5.5 times compared to the PbTe photodetector. Moreover, the operating frequency band of the 2DEG photodetector is broadened to 22 to 519 GHz. By comparing a range of detection performance metrics with other RT THz photodetectors, our 2DEG photodetector demonstrates greatly superior performance.

Finally, RT THz detectors hold great promise for expanding THz technology into practical applications due to their operational convenience and cost-effectiveness compared to cryogenically cooled alternatives. However, state-of-the-art detection strategies are still constrained by the trade-offs between sensitivity, response range, response speed, fabrication complexity, and scalability for large arrays. We introduce a broadband, high-sensitivity, rapid response RT THz detection technology. It is worth mentioning that the proposed photodetector features a simple MSM structure, which reduces large-scale fabrication complexity and makes it easier to maintain device consistency. Given that the high-quality wafer-scale CdTe/PbTe 2DEG heterojunction samples can be grown on Ge (111) or Si (111) wafers by MBE [42,43], it is promising to achieve high-resolution and real-time imaging by integrating focal plane arrays of the 2DEG photodetector with readout integrated circuits using flip chip bonding technology. The CdTe/PbTe materials used in the proposed THz detector offer excellent availability and compatibility with standard semiconductor processing. Consistent material growth quality is crucial for achieving uniformity in responsivity and detectivity. Both CdTe and PbTe are well-established compound semiconductors with mature growth techniques, enabling the production of high-quality crystals with controlled doping and structural precision. Furthermore, the ability to grow these materials on Si and Ge substrates further enhances their integration potential with existing semiconductor technologies, reducing production costs and simplifying large-scale array manufacturing.

## Materials and Methods

### Device fabrication

Utilizing MBE technology, two 500-nm films were grown on 2 BaF_2_ substrates. One was followed by the growth of a 100-nm-thick CdTe film to form the CdTe/PbTe heterojunction. The photoresist was evenly spined onto the smooth material surfaces, followed by ultraviolet lithography technology to pattern the sample surfaces. After lithography, 2 sample surfaces in the designed photo-sensitive area (*w* = 5 μm, *a* = 30 μm) and the same metal electrode contact area were covered by photoresist, leaving the rest exposed. The exposed semiconductor films were removed by inductively coupled plasma (ICP) etching technology. The residual photoresist was then cleaned. Another lithography process was performed to pattern the butterfly-shaped electrode area. To achieve good ohmic contact between the CdTe/PbTe semiconductor and the metal electrode, we first evaporated 100 nm of Cu and annealed it at 150 °C for 10 min, allowing Cu atoms to diffuse into the 2DEG channel and form an ohmic contact. Finally, Cr (10 nm)/Au (200 nm) was evaporated on 2 devices to form the electrodes, followed by facilitating wire bonding and integration into the external test circuit.

### Material characterizations

The surface morphology of the PbTe semiconductor was captured using an AFM (Innova, Bruker). SEM (SU8010) was used to provide the cross-sectional morphology of the heterojunction. The crystalline quality of the material was characterized by XRD (Empyrean) using a Cu target x-ray (*λ* = 0.15406 nm). HRTEM (JEM-2100F) provided the cross-sectional image at the heterojunction interface of CdTe/PbTe grown along the [111] direction.

### Photoelectric performance

We employed an RT THz response performance testing system to characterize the photoelectric performance of the photodetectors. The THz wave sources (VDI: 165 to 173 GHz, 330 to 346 GHz, and 495 to 519 GHz) and microwave source (Agilent E8257D: 20 to 40 GHz) are electrically modulated to serve as the reference signal for a lock-in amplifier. An SR 570 pre-amplifier is used to provide a biased voltage onto the photodetectors and amplify the electric signal, which is then read out through an SR 830 lock-in amplifier. Additionally, we tested the current-noise spectra of the photodetectors using a system composed of an electromagnetic shielding box, a FEMTO DLPCA-200 low-noise pre-amplifier, and an SRS SR770 spectrum analyzer.

## Supplementary Materials

Supplementary 1 Notes S1 to S5 Figs. S1 to S5 Table S1 References [53,54]
