# Supplementary material for: High-Performance Room-Temperature Terahertz Photodetection Using 2-Dimensional Electron Gas Channel Transport
Source: Research (Wash D C). 2025 Mar 26;8:0656. doi: 10.34133/research.0656 (PMC11938713; doi:10.34133/research.0656)
Supplement: Supplementary 1 — Notes S1 to S5 Figs. S1 to S5 Table S1 References [53,54] [file research.0656.f1.docx]

**Supplementary Materials**

**High-performance room-temperature terahertz photodetection using 2DEG Channel Transport**

**Mengjuan Liu,^1,^**^†^ **Yongzhen Li,^2,3,^**^†^ **Ziyang Ren,^1^ Yao Wang,^1^ Haiming Zhu,^1^ Qinxi Qiu,^2^ Nasir Ali,^5^ He Zhu,^3,4^ Jiaqi Zhu,^3,4,*^ Weien Lai,^6,*^ Zhiming Huang,^2,3,4,*^ Huizhen Wu^1,*^**

*^1^Zhejiang Province Key Laboratory of Quantum Technology and Devices, School of Physics, and State Key Laboratory of Silicon and Advanced Semiconductor Materials, Zhejiang University, Hangzhou 310058, China*

*^2^State Key Laboratory of Infrared Physics, Shanghai Institute of Technical Physics, Chinese Academy of Sciences, Shanghai 200083, China*

*^3^University of Chinese Academy of Sciences, Chinese Academy of Sciences, Beijing 100049, China*

*^4^Hangzhou Institute for Advanced Study, University of Chinese Academy of Sciences, Hangzhou, Zhejiang 310024, China*

*^5^Computing Research Center for Intelligent Manufacturing, Zhejiang Lab, Yuhang District, Hangzhou, Zhejiang, 311121 China*

*^6^School of Instrument Science and Opto-electronics Engineering, Hefei University of Technology, Hefei, Anhui, 230009, China*

*^*^jiaqizhu@ucas.ac.cn*

*^*^wnlai@hfut.edu.cn*

*^*^zmhuang@mail.sitp.ac.cn*

*^*^hzwu@zju.edu.cn*

^†^These authors contributed equally to this paper.

**Note S1.** **Hall effect measurement**


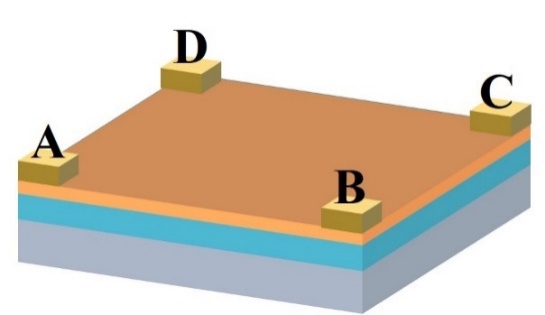


**Fig. S1 Schematic diagram of Hall effect measurement**

We applied the van der Pauw method to measure the Hall effect of the CdTe/PbTe heterojunction and the PbTe materials, as shown in Fig. S1. The four electrodes are located at the corner edges of the square film, whose sizes are much smaller than that of the films.

**(1) Resistivity**

First, a constant current *I*_AB_ is applied between A and B, and then a voltage *V*_CD_ can be obtained between C and D. Thus, a resistance can be defined as:

$$\begin{aligned} R_{AB,CD}=\frac{\left| V_{\mathrm{CD}} \right|}{I_{\mathrm{AB}}}\#\left( S1 \right) \end{aligned}$$

Next, a constant current *I*_BC_ is applied between B and C, and similarly, another voltage *V*_DA_ can be obtained between D and A. Another resistance can be defined as:

$$\begin{aligned} R_{BC,DA}=\frac{\left| V_{\mathrm{DA}} \right|}{I_{\mathrm{BC}}}\#\left( S2 \right) \end{aligned}$$

The resistivity *ρ* and the above two resistances follow the equation:

$$\begin{aligned} \exp\left( -\pi\frac{d}{\rho}R_{AB,CD} \right)+\exp\left( -\pi\frac{d}{\rho}R_{BC,DA} \right)=1\#\left( S3 \right) \end{aligned}$$

where *d* is the film thickness. We can solve this equation via introducing a symmetry factor *f*:

$$\begin{aligned} \rho=\frac{\pi}{\ln2}d\frac{R_{AB,CD}+R_{BC,DA}}{2}f\#\left( S4 \right) \end{aligned}$$

where *f* can be acquired using an interpolation method:

$$\begin{aligned} \cosh\left\{ \frac{\ln2}{f}\cdot\frac{\frac{R_{AB,CD}}{R_{BC,DA}}-1}{\frac{R_{AB,CD}}{R_{BC,DA}}+1} \right\}=\frac{1}{2}\exp\left( \frac{\ln2}{f} \right)\#\left( S5 \right) \end{aligned}$$

If the four electrodes are perfectly symmetrical, i.e. the two resistances are equal, we can obtain that *f* = 1.

**(2) Hall coefficient**

First, a constant magnetic field with an intensity of *H* is applied vertically across the film. Next, a constant current *I*_AC_ is applied between A and C, and then a voltage *V*_BD_ can be obtained between B and D. A Hall coefficient *R*_H1_ is defined as:

$$\begin{aligned} R_{H1}=\frac{V_{\mathrm{BD}}d}{I_{\mathrm{AC}}H}.\#\left( S6 \right) \end{aligned}$$

Similarly, a constant current *I*_BD_ is applied between B and D, and then a voltage *V*_AC_ can be obtained between A and C. Another Hall coefficient *R*_H2_ is also defined as:

$$\begin{aligned} R_{H2}=\frac{V_{\mathrm{AC}}d}{I_{\mathrm{BD}}H}.\#\left( S7 \right) \end{aligned}$$

If the film is with a good uniformity, the difference between the two coefficients should be within 10%. In this case, the true Hall coefficient is:

$$\begin{aligned} R_{H}=\frac{R_{H1}+R_{H2}}{2}.\#\left( S8 \right) \end{aligned}$$

By continuously changing *H* within a certain range, a curve of *V_d_*/*I*-*H* is obtained. According to Eqs. S6 and S7, *V_d_*/*I*-*H* exhibits a linear trend with a slope of *R*_H_. Hence, *R*_H_ can be obtained. When the slope is positive, the electrical conduction of the film is p-type, otherwise it is n-type.

**(3) Carrier concentration**

The majority carrier concentration *n* can be obtained by:

$$\begin{aligned} n=\frac{1}{R_{H}q}.\#\left( S9 \right) \end{aligned}$$

**(4) Carrier mobility**

When no magnetic field is applied, a resistivity *ρ*_0_ is obtained. Then the mobility can be obtained by:

$$\begin{aligned} \mu=\frac{R_{H}}{\rho_{0}}.\#\left( S10 \right) \end{aligned}$$

**Note S2. The calculation process of the lattice constants**

**
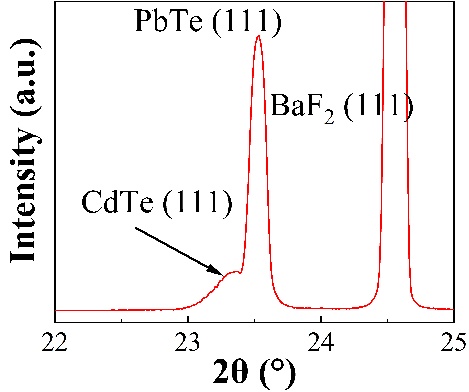
**

**Fig. S2 The XRD pattern of the CdTe/PbTe heterojunction**

X-ray diffractometer was employed to test the CdTe/PbTe heterojunction using Cu K_α_ radiation. The results given in Fig. S2 show that the diffraction spectrum contains diffraction peaks of PbTe, CdTe, and BaF_2_ along the [111] crystal orientations. This indicates that single-crystal epitaxy of PbTe (111) and CdTe (111) has been achieved on the BaF_2_ substrate. Using Bragg's Law and the interplanar spacing formula can calculate the lattice constants *a*_0_ of the materials:

$$\begin{aligned} 2dsin\theta=n\lambda\#\left( S11 \right) \end{aligned}$$

$$\begin{aligned} d=\frac{a_{0}}{\sqrt{h^{2}+k^{2}+l^{2}}}\#\left( S12 \right) \end{aligned}$$

Here, *d* is the interplanar spacing, *λ* is X-ray wavelength.

**Note S3. The origin of the EIW effect**


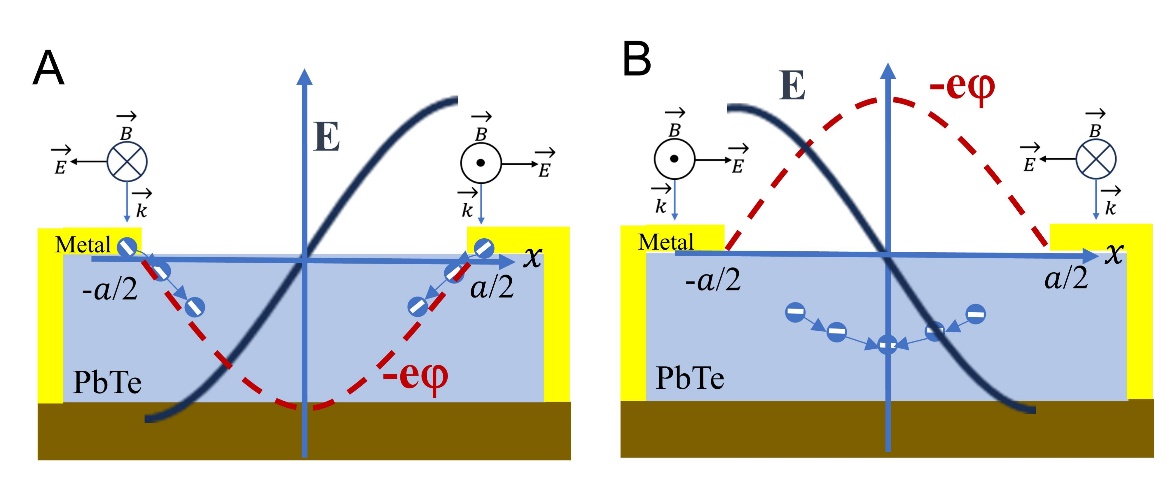


**Fig. S3 The electromagnetic transverse magnetic (TM) wave has an anti-symmetric electric field (blue solid curve) that varies with the exposed ends of the photosensitive material as periodic boundaries. The symmetric potential well during the first half period (red dashed curve in A) and the symmetric potential barrier during the second half period (red dashed curve in B).**

We are presently engaged in theoretical analysis of the detection mechanism of the terahertz detector employing an MSM structure [34,35]. Upon incidence of a TM-mode electromagnetic wave on the MSM structure depicted in Fig. S3, an anti-symmetric electric field (blue solid curve) appears parallel to the device surface along the *x*-direction. In first half period, the electrons in the metal are emitted into the semiconductor driven by Lorentz force *F* = *q*(*E* *+ v* × *B*) as shown in Fig. S3(A). Because of the intrinsic relation of the electric potential and electric field, there is an EIW formed. The electrons are confined in the EIW due to its low potential energy. In the following second half period when the anti-symmetric electric field in Fig. S3(B) appears, the injected electrons in the semiconductor are decelerated but do not go back into the electrodes. The equivalent potential barrier helps to retain the emitted electrons. Finally, the non-equilibrium electrons are stored in the semiconductor and can be read out by voltmeter at biased voltage. The electric and magnetic fields of the TM electromagnetic wave have the following forms:

$$\begin{aligned} E\left( x,y,z,t \right)=\left( E_{x}\hat{x}+E_{y}\hat{y}+E_{z}\hat{z} \right)e^{i\left( k_{z}z-\omega t \right)}\#\left( S13 \right) \end{aligned}$$

$$\begin{aligned} B\left( x,y,z,t \right)=\left( B_{x}\hat{x}+B_{y}\hat{y}+B_{z}\hat{z} \right)e^{i\left( k_{z}z-\omega t \right)}\#\left( S14 \right) \end{aligned}$$

In this context, *E_y_*_​_, *B_x_*_​_, and *B_z_​* are zero. The anti-symmetric electric field in the *x*-direction can be expressed as:

$$\begin{aligned} E_{x}=E_{1}\sin\left( k_{x}x \right)\#\left( S15 \right) \end{aligned}$$

Given *E* = -∇*ϕ* and considering only the range of first-order reciprocal lattice vectors, the anti-symmetric electric field in the MSM structure induces a symmetrically distributed potential, as shown in Fig. S3, which can be expressed in the following form:

$$\begin{aligned} \varphi=\varphi_{1}\cos\left( \frac{\pi}{a}x \right)e^{i\left( k_{z}z-\omega t \right)}+C\#\left( S16 \right) \end{aligned}$$

According to the D’Alembert equation:

$$\begin{aligned} \nabla^{2}\varphi-\varepsilon_{0}\mu_{0}\frac{\partial^{2}\varphi}{\partial^{2}t}=-\frac{\rho}{\varepsilon_{0}}\#\left( S17 \right) \end{aligned}$$

and considering the integration of *ρ* in the direction of the *x* and *z* axes for half phase period (*ε*_0_ and *μ*_0_ are the permittivity and permeability, respectively, and *ρ* is the charge density generated), then we can get the averaged charge density:

$$\begin{aligned} \bar{\rho}=\frac{4\varepsilon_{0}aE_{1}}{\pi^{3}qd\sqrt{\varepsilon_{r}}}\sqrt{\left( \frac{\pi}{a} \right)^{2}-k_{0}^{2}}\left[ 1-exp\left( -d\sqrt{\varepsilon_{r}}\sqrt{\left( \frac{\pi}{a} \right)^{2}-k_{0}^{2}} \right) \right]\#\left( S18 \right) \end{aligned}$$

The relation between the electric field intensity *E*_0_ of the light and the incident light power *P* is given by *E*_0_ = *P*/*cq*. Furthermore, the amplitude of the electric field *E*_1_ in the structure can be written as *E*_1_ = *ηE*_0_, where $\eta=\left| \frac{\varepsilon\left( \omega\right)\sqrt{{\varepsilon_{0}\mu_{0}k}_{0}^{2}-{(\pi/a)}^{2}}}{\sqrt{{{\varepsilon\left( \omega\right)\varepsilon}_{0}\mu_{0}k}_{0}^{2}-{(\pi/a)}^{2}}} \right|$ is the electric field enhancement factor in the metal spacing and is determined by boundary conditions. And *ε*(*ω*) is the relative permittivity of the metal at the special frequency, which can be calculated by Drude model $\varepsilon\left( \omega\right)=\varepsilon_{\infty}-\frac{\omega_{p}^{2}}{\omega(\omega+i\omega\tau)}$. Finally, in terms of $\bar{\rho}=\Delta n(-q)$, the variation of electron concentration can be derived in the semiconductor as follows:

$$\begin{aligned} \Delta n=\frac{4\varepsilon_{0}a\eta P}{\pi^{3}q^{2}cd\sqrt{\varepsilon_{r}}}\sqrt{\left( \frac{\pi}{a} \right)^{2}-k_{0}^{2}}\left[ 1-exp\left( -d\sqrt{\varepsilon_{r}}\sqrt{\left( \frac{\pi}{a} \right)^{2}-k_{0}^{2}} \right) \right]\#\left( S19 \right) \end{aligned}$$

where *a* is the length of the photosensitive region, *q* is the unit charge, *c* is the speed of light, *d* is the thickness of the photosensitive material, *ε*_r_ is the relative permittivity of the photosensitive material, and *k*_0_ is the wave vector of light. We introduce the photoconductive gain Γ_e_ by drawing on classical photoconductivity theory. It depends on the ratio of the minority carrier lifetime to the transit time in the photoconductive device: $\Gamma_{e}={\tau_{e}}/{\tau_{d}}$, where *τ*_e_ is the minority carrier lifetime in the semiconductor material, and *τ*_d_ is the transit time of excess carriers under the influence of the bias electric field. Therefore, the change in electron concentration in the semiconductor material of the device should be:

$$\begin{aligned} \Delta n=\frac{4\varepsilon_{0}a\eta P\Gamma_{e}}{\pi^{3}q^{2}cd\sqrt{\varepsilon_{r}}}\sqrt{\left( \frac{\pi}{a} \right)^{2}-k_{0}^{2}}\left[ 1-exp\left( -d\sqrt{\varepsilon_{r}}\sqrt{\left( \frac{\pi}{a} \right)^{2}-k_{0}^{2}} \right) \right]\#\left( S20 \right) \end{aligned}$$

The change in carrier concentration in a semiconductor material results in a change in device resistance.

When the semiconductor is the CdTe/PbTe heterojunction with 2DEG, the non-equilibrium electrons would drift rapidly into the 2DEG channel due to the band bending at the heterojunction interface. In the high-density and high-mobility 2DEG transport channel, the non-equilibrium electrons can be collected efficiently and quickly.

**Note S4.** **The band bending at the heterojunction interface**


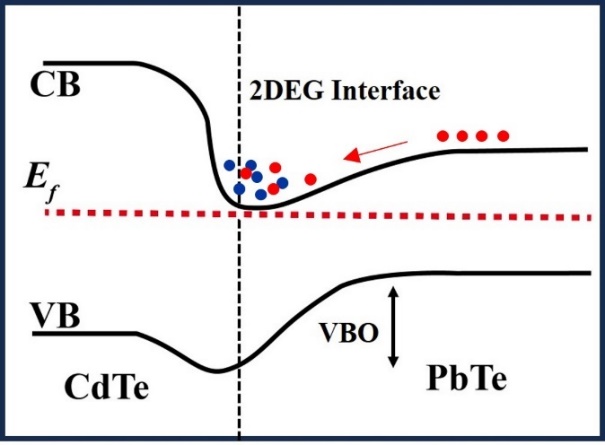


**Fig. S4 The energy band** **profile of the CdTe/PbTe heterojunction**

**Note S5. The optimal NEPs of two photodetectors.**

**
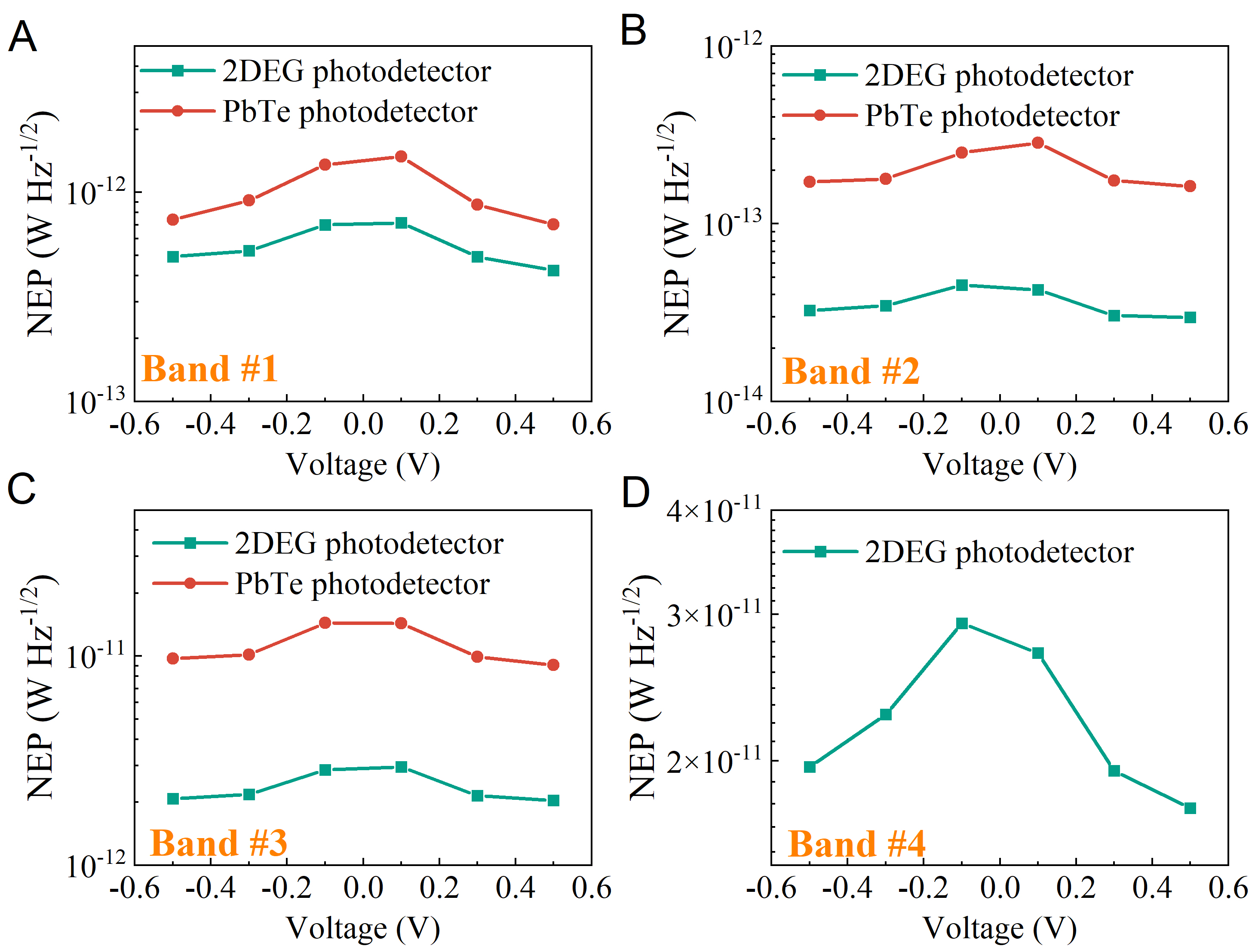
**

**Fig. S5 (A-D) The NEPs of the devices across four frequency bands at various biased voltages.**

**Table S1.** **Performance comparison of the 2DEG photodetector with other state-of-the-art RT THz detectors**

| **Material** | **Mechanism** | **Operation frequency band (THz)** | **NEP (****W/Hz^1/2^)** | **Response**  **time** | **Ref.** |
| --- | --- | --- | --- | --- | --- |
| Golay cell | Bolometer effect | 0.04-20 | 1.4 × 10*^−^*^10^ | 30 ms | [39] |
| Graphene | Ballistic rectifier | 0.1-0.4 | 3.4 × 10^-11^@0.345 THz | - | [40] |
| Bi_2_Se_3_ | EIW+SPP | 0.04-0.3 | 3.6 × 10^-13^@0.12 THz | 66 μs | [41] |
| GaN/AlGaN | HEMT | 0.1-1.1 | 3.7 × 10^-12^@0.65 THz | - | [20] |
| Si | FET | 0.63 | 1 × 10^-11^ | - | [17] |
| PtTe_2_ | Topological effect | 0.12 | 1 × 10^-11^ | 20 μs | [42] |
| PdTe_2_ | Photogalvanic effect | 0.04-0.3 | 2 × 10^-12^@0.12 THz | 1 μs | [43] |
| InSb | SPP | 0.36 | 1.5 × 10^-13^ | 2.7 μs | [21] |
| HgTe/CdTe | PTE | 0.1 | 3.2 × 10^-12^ | 2.5 μs | [44] |
| BP | PTE | 0.04 | 1 × 10^-11^ | 4 μs | [45] |
| WR15 ZBD | SBD | 0.05-0.075 | 3.4 × 10^-12^@0.075 THz | < 1 µs | [46] |
| NbIrTe_4_ | Topological effect | 0.1 | 5.1 × 10^−11^ | 10.8 µs | [31] |
| PtSe_2_ | Intraband transition | 0.1-0.3 | 3.8 × 10^−11^@0.3 THz | 1 µs | [33] |
| EuSn_2_As_2_ | PTE+Anisotropic scattering | 0.02-0.03 | 3 × 10^−11^@0.03 THz | 16 µs | [47] |
| ZrGeSe | PTE | 0.26 | 1.5 × 10^−10^ | 8.3 µs | [48] |
| **CdTe/PbTe** | **EIW+2DEG transport** | **0.022-0.519** | **3.0 × 10^-14^@0.166 THz** | **6.7 µs** | **This work** |
